# USP7 Regulates Cytokinesis through FBXO38 and KIF20B

**DOI:** 10.1038/s41598-019-39368-y

**Published:** 2019-02-25

**Authors:** Anna Georges, Etienne Coyaud, Edyta Marcon, Jack Greenblatt, Brian Raught, Lori Frappier

**Affiliations:** 1 Department of Molecular Genetics, University of Toronto, Toronto, Ontario Canada; 2 The Princess Margaret Cancer Centre, University Health Network, Toronto, Ontario M5G 1L7 Canada; 3 Donnelly Centre, University of Toronto, Toronto, Canada; 4 Department of Medical Biophysics, University of Toronto, Toronto, Ontario Canada

## Abstract

The ubiquitin specific protease 7 (USP7 or HAUSP) is known to regulate a variety of cellular processes by binding and deubiquitylating specific target proteins. To gain a more comprehensive understanding of its interactions and functions, we used affinity purification coupled to mass spectrometry to profile USP7 interactions. This revealed a novel interaction with FBXO38, a poorly characterized F-box protein. We showed that USP7 stabilizes FBXO38 dependent on its catalytic activity by protecting FBXO38 from proteasomal degradation. We used a BioID approach to profile the protein interactions (and putative functions) of FBXO38, revealing an interaction with KIF20B, a Kinesin-6 protein required for efficient cytokinesis. FBXO38 was shown to function independently from an SCF complex to stabilize KIF20B. Consequently, depletion of either FBXO38 or USP7 led to dramatic decreases in KIF20B levels and KIF20B at the midbody, which were manifested in cytokinetic defects. Furthermore, cytokinetic defects associated with USP7 silencing were rescued by restoring FBXO38 or KIF20B. The results indicate a novel mechanism of regulating cytokinesis through USP7 and FBXO38.

## Introduction

The ubiquitin specific protease 7 (USP7), also known as HAUSP (Herpesvirus Associated Ubiquitin Specific Protease), is a deubiquitylating enzyme (DUB) that removes ubiquitin from specific target proteins, often resulting in their stabilization due to protection from proteasomal-mediated degradation. Due to its wide variety of substrates, USP7 has been found to be an important regulator of many cellular processes, including apoptosis, the cell cycle, gene expression, DNA damage responses and DNA replication^1–4^. USP7 misregulation is also associated with several cancers^5–9^. For example, USP7 overexpression has been shown to correlate with poor prognosis in lung and ovarian cancer and with tumor aggressiveness in prostate cancer^7,9,10^. However, both overexpression and downregulation of USP7 have been observed in breast and colon cancer^6,11–13^. The association of USP7 with cancer has sparked a major interest in the development of USP7 inhibitors as anti-cancer therapies^14–20^.

USP7 was first identified as a binding partner of the herpes simplex virus 1 (HSV-1) ICP0 protein, and later shown to be the target of multiple proteins from several different viruses, particularly herpesviruses^21–33^. The first cellular functions identified for USP7 were in the regulation of the p53 pathway. Studies showed that, upon DNA damage induction, USP7 directly deubiquitylates and stabilizes the p53 tumor suppressor protein^34,35^. Alternatively, under normal cellular conditions USP7 can act as a negative regulator of p53 by deubiquitylating and stabilizing the dominant p53 E3 ubiquitin ligases Hdm2 and HdmX^36,37^. Since then, USP7 has been shown to deubiquitylate and stabilize numerous other proteins with a variety of functions^38–44^. In addition to cleaving polyubiquitin chains that target proteins for degradation, USP7 can cleave monoubiquitin to alter protein localization or function. For example, USP7 cleaves monoubiquitin from histone H2B to impact gene expression and similarly removes monoubiquitin from FOXO4 to regulate its transcriptional activity^5,27,45,46^. Finally, USP7 has also been found to negatively regulate promyelocytic leukemia (PML) proteins and nuclear bodies through a mechanism independent of its deubiquitylating activity^47^.

A number of reports have demonstrated the importance of USP7 in regulating progression through the cell cycle. First, studies have shown that depletion of USP7 in cancer cells is positively correlated with a G1 arrest, which can be triggered in some cases by p53 accumulation^14,48,49^. In other cases, USP7 depletion may result in G1 arrest due to destabilization of USP7 targets UHRF1 and Chk1, which are required for G1/S transition^43,50–54^. We have previously shown that USP7 also promotes late S phase and G2 progression by facilitating unloading of the Minichromosome Maintenance protein (MCM) complex from chromatin during DNA-replication termination^55^. Further supporting its role in DNA replication, USP7 was shown to be a SUMO deubiquitylase that functions to maintain high concentrations of SUMOylated factors at replication forks, which is necessary for replication-fork progression^56^. In addition, USP7 was recently found to stabilize Geminin; a protein that inhibits replication origin licensing by Cdt1^13^. USP7 also regulates early mitotic progression by stabilizing the mitotic checkpoint protein CHFR, which is responsible for delaying entry into metaphase in response to mitotic stress^49,57,58^.

The numerous roles of USP7 stem from its ability to specifically bind multiple target proteins. USP7 uses two different binding pockets to recognize its target proteins, both of which are distinct from its central catalytic domain^4^. The first identified binding pocket is within the N-terminal TRAF domain (NTD). Many USP7 targets bind this pocket including p53, Hdm2, HdmX, MCM-BP, Epstein-Barr virus EBNA1 and Kaposi’s sarcoma associated herpesvirus (KSHV) vIRF1 and vIRF4^24,26,33,55,59–61^. Structures and mutational analyses of these interactions identified a P/A/ExxS motif in all of these targets that is responsible for the USP7 interaction, and showed that USP7 amino acids D164 and W165 are essential for mediating these interactions^26,33,55,59–61^. More recently, a second binding pocket was identified in USP7, found within one of the ubiquitin-like structures (Ubl2) in the C-terminal domain (CTD). This pocket is bound by GMP synthetase (GMPS), DNMT1, UHRF1 and the HSV-1 protein ICP0 through KxxxK motifs that contact USP7 residues D762 and D764^62–66^.

The above discoveries of USP7 functions have come from studies on the specific target proteins. To gain a more comprehensive understanding of USP7 interactions and potentially identify novel targets, we used an affinity purification-mass spectrometry (AP-MS) approach to profile interactions with USP7. This identified a novel interaction with a poorly characterized F-box protein, FBXO38, whose stability is dependent on USP7. We then used the BioID proteomics method to identify interactors and potential functions of FBXO38, revealing an interaction with KIF20B, a key regulator of cytokinesis. We show that FBXO38 has an important role in stabilizing KIF20B, thereby contributing to cytokinesis, and that USP7 indirectly regulates KIF20B and cytokinesis through FBXO38. Therefore, we have identified previously unknown roles for USP7 and FBXO38 in the regulation of cytokinesis.

## Results

### USP7 interacts with and stabilizes FBXO38

To characterize new functions of USP7 through the identification of novel interactors, we performed affinity purification coupled to mass spectrometry (AP-MS) on FLAG-tagged USP7 that was expressed in AGS gastric carcinoma cells. To keep USP7 levels low, potentially avoiding artifacts of overexpression, we delivered FLAG-USP7 in an adenovirus expression system at low multiplicity of infection that resulted in expression in ~70% of the cells at levels close to endogenous (Supplementary Fig. S1A,B). FLAG-tagged β-galactosidase (β-gal) was used as a negative control and was similarly delivered. Bait proteins were recovered on anti-FLAG resin and similar recoveries of β-gal and USP7 were confirmed by Western blot (Supplementary Fig. S1C). Co-purifying proteins were identified by LC-MS/MS of tryptic peptides. Table 1 shows the peptide recovery (total spectral counts) of USP7 specific interactors, determined by comparison of recoveries with β-gal as well as to average values in the Contaminent Repository for Affinity Purification database http://www.crapome.org/; a repository of results of over 400 AP-MS experiments^67^. The complete data for USP7 and β-gal are provided in Supplementary Table S1. Previously characterized USP7 interactors, including GMPS, the ATM-dependant phosphatase PPM1G, DNA methyltransferase DNMT1 and the E3 ligase TRIP12 were among the specifically recovered proteins, providing validation of our method^27,46,52,53,62,64,68–72^. We also identified a number of uncharacterized USP7 interactors, including the F-box protein FBXO38, the deubiquitylating enzyme USP11, DEAD-box helicase DDX24, Peptidylprolyl Isomerase PPIL4, DEAH-Box Helicase DDX40, Lamina-associated polypeptide TMPO and the transcription elongation factor TCEAL4.

**Table 1:** Affinity Purification-Mass Spectrometry Results for FLAG-USP7.

Identified proteins	Total Spectral Counts			Length (amino acids)	Peptide counts/length
	FLAG-USP7	FLAG-β GAL	CRAPome average		
USP7	2307	0		1102	2.093
GMPS	303	0	3.6	693	0.437
USP11	159	0	3.2	963	0.165
TRIP12	133	0	4.3	1992	0.067
DNMT1	188	0	3.7	1616	0.116
DDX24	126	0	3.4	859	0.147
PPIL4	80	0	4.7	492	0.163
DHX40	68	0	2	779	0.087
PPM1G	49	0	2	546	0.090
TMPO	68	0	13.6	694	0.098
FBXO38	54	0	0	1188	0.045
TCEAL4	24	0	2	215	0.112

**Figure 1:**
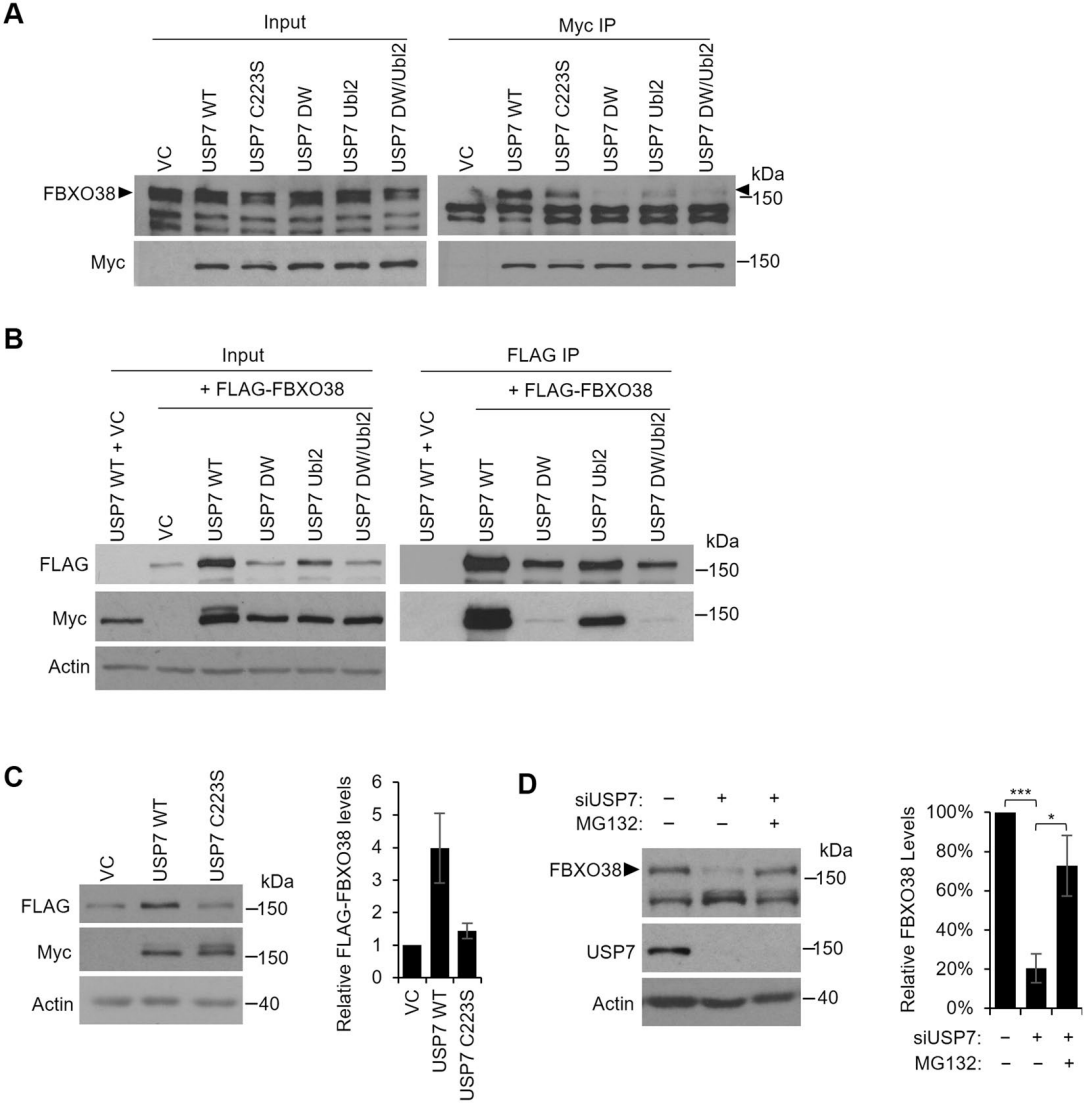
USP7 interacts with and stabilizes FBXO38. (**A**) AGS cells were transfected with a plasmid expressing myc-tagged USP7 WT, the USP7 catalytic mutant (C223S), the USP7 NTD pocket mutant (DW), the USP7 Ubl2 pocket mutant (Ubl2), the USP7 double pocket mutant (DW/Ubl2) or an empty vector control (VC). Myc-USP7 constructs were immunoprecipitated with anti-myc antibody and recovered proteins were analyzed by Western blotting using antibodies against myc and endogenous FBXO38. The band corresponding to FBXO38 is indicated with arrow heads. (**B**) HEK293T cells were transfected with a plasmid expressing myc-tagged USP7 WT or co-transfected with plasmids expressing FLAG-tagged FBXO38 and each of the indicated myc-tagged USP7 plasmids. FBXO38 was immunoprecipitated using anti-FLAG M2 resin and recovered proteins were analyzed by Western blotting using antibodies against FLAG and myc. (**C**) HEK293T cells were co-transfected with plasmids expressing FLAG-tagged FBXO38 and either myc-USP7 WT, C223S or an empty vector control (VC). Cells were harvested 48 h post transfection and cell lysates were analyzed by Western blotting using antibodies against FLAG, Myc and actin. Quantification of the FLAG-FBXO38 bands (normalized to actin) from two independent experiments is shown on the right. (**D**) AGS cells were transfected with an siRNA targeting USP7 (+) or a negative control siRNA (−) followed by treatment with the MG132 proteasome inhibitor (+) or DMSO as a negative control (−) for 12 hours. Cell lysates were analyzed by Western blotting using antibodies against FBXO38, USP7 and actin. Quantification of the FBXO38 bands (normalized to actin) from three independent USP7 silencing experiments are shown on the right. ***P < 0.001.

In this study, we focused on the USP7-FBXO38 interaction, which was also detected by Sowa *et al*.^73^ but not characterized. F-box proteins typically form Skp1-cullin- F-box (SCF) E3 ubiquitin ligase complexes. However, the only reported function for FBXO38 is a co-activator of the KLF7 transcription factor and this is independent of an SCF complex^74–76^. To investigate the significance of the USP7-FBXO38 interaction, we first validated the interaction by transiently expressing myc-tagged USP7 in AGS cells, performing myc immunoprecipitation and immunoblotting for endogenous FBXO38 (Fig. 1A; full length Western blots for all figures are provided in the Supplementary Fig. S2). USP7 but not empty plasmid recovered FBXO38. The interaction was also verified by co-expressing myc-USP7 and FLAG-FBXO38 in 293T cells, followed by FLAG IP (Fig. 1B). Myc-USP7 was efficiently recovered in samples containing FLAG-FBXO38 but not empty FLAG plasmid. We then used these expression systems to determine how USP7 binds FBXO38. We and others previously defined two USP7 binding pockets, one in the N-terminal TRAF domain (NTD) that is disrupted by D164A,W165A mutations (referred to here as DW) and a second in the Ubl2 ubiquitin-like domain that is disrupted by D762R,D764R mutations (referred to here as Ubl2)^26,59–62^. We compared recovery of endogenous FBXO38 with myc-USP7 containing WT sequence or DW or Ubl2 mutations (individually and in combination) or mutation in the USP7 catalytic cysteine (C223S) (Fig. 1A). Both the DW and Ubl2 mutations decreased FBXO38 recovery to some degree, with the DW mutation having the greater impact. The effect of these binding pocket mutations was also assessed by co-expression with FLAG-FBXO38 in 293T cells followed by FLAG IP (Fig. 1B). The Ubl2 mutant of USP7 was clearly recovered with FLAG-FBXO38, whereas the DW and DW/Ubl2 mutations abrogated the interaction. Together the results indicate that, while both the NTD and Ubl2 pockets contribute to FBXO38 binding, the NTD binding pocket is the major binding site.

Another interesting finding of the experiment in Fig. 1B, was that the level of FLAG-FBXO38 was significantly increased when it was co-expressed with myc-USP7 (compare lanes 2 and 3) and that the degree of increase of FLAG-FBXO38 paralleled the degree of binding of the USP7 mutants; DW and DW/Ubl2 mutants that did not bind FBXO38 did not upregulate it and the Ubl2 mutant that had decreased binding had an intermediate effect on FBXO38 levels. These results suggest that USP7 binding to FBXO38 stabilizes it, possibly by deubiquitylation. To investigate this further, we asked whether upregulation of FBXO38 required the catalytic activity of USP7. Comparison of FLAG-FBXO38 levels co-expressed with WT or catalytically inactive (C223S) USP7 or empty plasmid showed that the catalytic activity of USP7 was required to upregulate FBXO38, consistent with FBXO38 stabilization by deubiquitylation (Fig. 1C). We also tested whether silencing of USP7 with siRNA affected the level of endogenous FBXO38 and found a dramatic reduction in FBXO38 upon USP7 depletion (Fig. 1D). To determine if this loss of FBXO38 was due to proteasomal-mediated degradation, we repeated the USP7 silencing in the presence of the MG132 proteasome inhibitor and found that this restored FBXO38 levels. Together the results suggest that USP7 binds and stabilizes FBXO38 through deubiquitylation, preventing its proteasomal-mediated degradation.

### Identification of KIF20B as an FBXO38 interactor stabilized by FBXO38 and USP7

**Figure 2:**
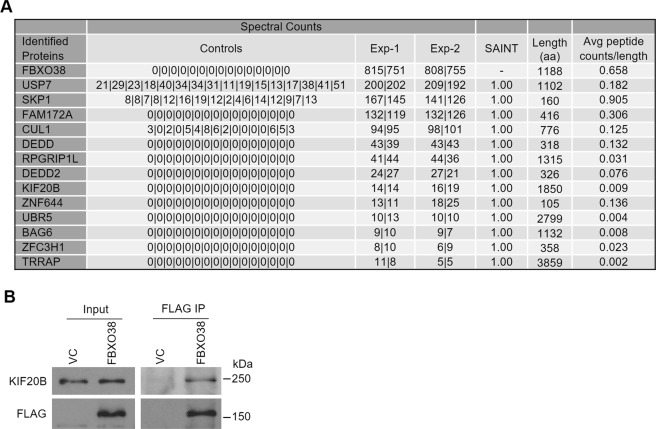
Identification of KIF20B as an interactor of FBXO38. (**A**) Expression of FLAGBirA*–FBXO38 or FLAGBirA* negative control from integrated cassettes in 293 T-REx cells was induced with tetracycline in the presence of 50 µM biotin for 24 hrs. Bioitinylated proteins were purified on Streptavidin Sepharose beads and recovered proteins were identified by LC-MS/MS. Total spectral counts for two biological replicates (Exp-1 and -2) with two technical replicates each (separated by) are shown for high-confidence FBXO38 interactors (based on Bayesian False Discovery Rate (BFDR) <0.01). Spectral counts for cells expressing FLAGBirA* tag alone (Control) are shown for 16 experiments. The length of each protein (amino acid numbers) and spectral counts per length are also shown for each protein. (**B**) AGS cells were transfected with a plasmid expressing FLAG-tagged FBXO38 (FBXO38) or an empty vector control (VC) for 48 hours. FBXO38 was immunoprecipitated using anti-FLAG M2 resin and recovered proteins were analyzed by Western blotting using antibodies against FLAG and KIF20B, respectively.

Little is known about the functions of FBXO38, other than it can act as a transcriptional coactivator with KLF7. To more comprehensively identify FBXO38 interactions that could reflect its functions, we performed FBXO38 proximity-dependent biotin identification (BioID), expressing a FLAGBirA*-tagged version of FBXO38 at close to endogenous levels from an inducible stably integrated cassette in 293 T-REx cells and compared the proximal interactions to the FLAGBirA* negative control. Selected high-confidence interactors with FLAGBirA*–FBXO38 in two independent experiments are shown in Fig. 2A. The complete list of specific interactors and raw data are provided in Supplementary Table S2 and the raw data files were deposited in the MassIVE database (https://massive.ucsd.edu/; accession number MSV000082444). Interestingly, USP7 was the most prevalent FBXO38 interactor. SKP1 and CUL1, the two core components for the SCF complex were also among the top hits, indicating that FBXO38 can form an SCF complex. In addition, we identified several novel interactions, including one with the Kinesin Family member 20B (KIF20B), a plus-end-directed motor enzyme that is required for the completion of cytokinesis^77–80^.

The interaction between FBXO38 and KIF20B was validated in AGS cells by transient expression and IP of FLAG-FBXO38 and immunoblotting for endogenous KIF20B (Fig. 2B). To further confirm the specificity of the FBXO38-KIF20B interaction, we also compared the recovery of endogenous KIF20B and transiently expressed HA-KIF20B with unrelated FLAG-tagged proteins (USP11 and β-gal). In both cases, KIF20B was preferentially recovered with FLAG-FBXO38, despite the much higher expression levels of FLAG-USP11 and FLAG- β-GAL (Supplementary Fig. S3). In addition, we examined recovery of KIF20B in 400 published BioID analyses of 200 different FLAGBirA*-tagged bait proteins, performed using similar systems and protocol as for FBXO38 (Supplementary Table S3)^81–84^. KIF20B was only detected in duplicate samples for 6 out of 200 proteins (and with lower recovery than for FBXO38), supporting the high specificity of the FBXO38-KIF20B interaction.

**Figure 3:**
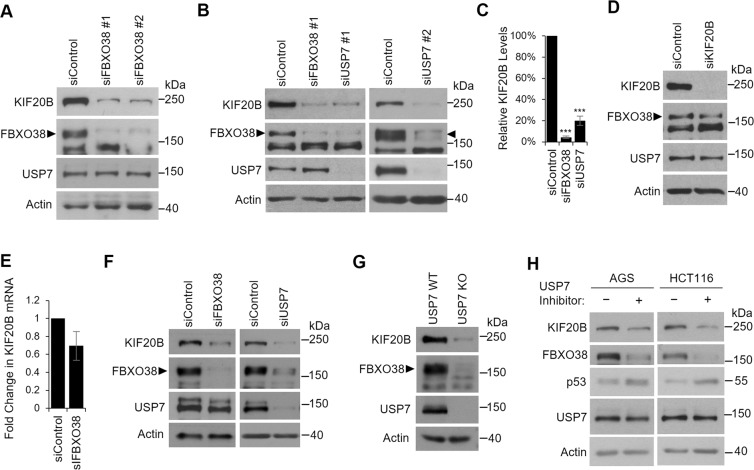
Depletion of FBXO38 or USP7 decreases KIF20B levels. AGS cells were transfected with two different siRNAs targeting FBXO38 (**A**) or USP7 (**B**) or with negative control siRNA (siControl) and whole cell lysates were analyzed by Western blotting using the indicated antibodies. Bands corresponding to FBXO38 are indicated with arrow heads. In AGS cells, an additional nonspecific band is also detected by this antibody running just below FBXO38. (**C**) KIF20B protein bands were quantified in three independent FBXO38 and USP7 silencing experiments in AGS cells and normalized to actin. The average values are shown relative the silencing control. ***P < 0.001. (**D**) AGS cells were transfected with an siRNA targeting KIF20B, and whole cell lysates were analyzed by Western blotting as in (**A**). (**E**) KIF20B mRNA levels were determined by RT-qPCR in AGS cells transfected with siRNA targeting FBXO38 or with negative control siRNA. The experiment was performed in triplicate and values normalized to actin. Average values and standard deviations are shown relative to the silencing control. (**F**) HCT116 cells were transfected with siRNA targeting FBXO38 or USP7 or with negative control siRNA (siControl) and whole cell lysates were analyzed by Western blotting as in (**A**). (**G**) Whole cell lysates of HCT116 USP7 KO or the WT parental cell line (USP7 WT) were analyzed by Western blotting as in (**A**). (**H**) AGS or HCT116 cells were treated with 5 µM of compound 4 (USP7 inhibitor) or an inactive enantiomer and harvested after 24 hours. Whole cell lysates were analyzed by Western blotting using the indicated antibodies.

The finding that FBXO38 can form an SCF complex, which would be expected to induce the degradation of specific targets, prompted us to examine whether FBXO38 regulated the levels of KIF20B. To this end, we silenced FBXO38 in AGS cells with two different siRNAs and examined effects on endogenous KIF20B (Fig. 3A). Surprisingly, both siRNAs resulted in a dramatic reduction in KIF20B levels (without affecting USP7 levels), suggesting that FBXO38 stabilized KIF20B, rather than destabilizing it as expected if FBXO38 was acting as part of the SCF complex. Since FBXO38 levels are controlled by USP7, we also examined the effect of USP7 silencing on KIF20B in AGS cells. As expected, USP7 depletion (with two different siRNAs) greatly decreased both FBXO38 and KIF20B levels (Fig. 3B). Quantification of these effects from multiple experiments showed a ~25 fold decrease in KIF20B upon FBXO38 silencing and ~5-fold decrease upon USP7 silencing (Fig. 3C). While both FBXO38 and USP7 control KIF20B levels, this effect was not reciprocal as silencing of KIF20B did not affect the levels of FBXO38 or USP7 (Fig. 3D). We also tested the possibility that the decrease in KIF20B upon FBXO38 silencing was due to loss of KIF20B transcripts (Fig. 3E). However, FBXO38 silencing only slightly decreased the level of KIF20B transcripts (~30% decrease) as opposed to the ~25-fold decrease seen at the protein level. Together the results support a model in which FBXO38 binds and stabilizes KIF20B and USP7 stabilizes KIF20B indirectly by stabilizing FBXO38.

To determine the generality of these effects, we repeated the FBXO38 and USP7 silencing experiments in HCT116 cells (Fig. 3F). Again we observed that depletion of either FBXO38 or USP7 greatly decreased KIF20B levels. HCT116 cells with USP7 knockout have been previously generated^37^, and our findings predict that these cells should have reduced levels of FBXO38 and KIF20B. A comparison of the levels of these proteins in HCT116 cells with and without the USP7 knockout confirmed that FBXO38 and KIF20B cells are considerably reduced in the absence of USP7 (Fig. 3G).

To verify that the regulation of FBXO38 and KIF20B levels by USP7 was due to the deubiquitylation activity of USP7, AGS and HCT116 cells were treated with a USP7 catalytic inhibitor (Compound 4, also known as AD04) recently developed by Harrison and colleagues^15^ or its inactive enantiomer (Fig. 3H). USP7 inhibition was confirmed by examining p53 levels, which are known to increase due to destabilization of the p53 E3 ubiquitin ligases, Hdm2 and Hdmx^15,36,85,86^. Consistent with the USP7 depletion and overexpression experiments, USP7 inhibition resulted in decreased levels of FBXO38 and KIF20B. This further supports our model that FBXO38 is stabilized by the deubiquitylation activity of USP7 which in turn stabilizes KIF20B.

### FBXO38 interacts with and stabilizes KIF20B by an SCF-independent mechanism

**Figure 4:**
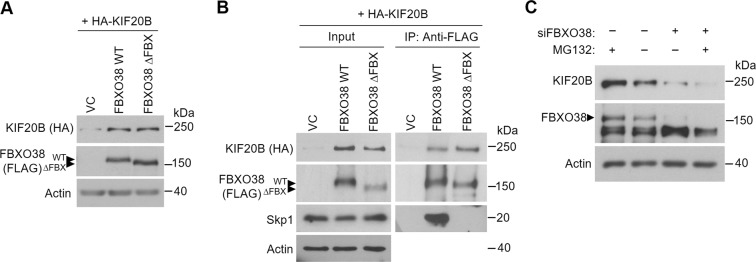
FBXO38 interacts with and stabilizes KIF20B by an SCF-independent mechanism. (**A**) AGS cells were cotransfected with plasmids expressing HA-tagged KIF20B and FLAG-tagged FBXO38 (WT), the FBXO38 F-box deletion mutant (∆FBX) or an empty vector control (VC). Cell lysates were analyzed by Western blotting using antibodies against FLAG and HA. (**B**) 293 T cells were cotransfected as in (**A**), followed by coimmunoprecipitation using anti-FLAG M2 resin. Recovered proteins were analyzed by Western blotting using antibodies against FLAG, HA and Skp1 and myc. Bands corresponding to FBXO38 WT or ∆FBX are indicated with arrow heads. (**C**) AGS cells were transfected with an siRNA targeting FBXO38 (+) or a negative control siRNA (−) followed by 12 hr treatment with the MG132 proteasome inhibitor (+) or DMSO (−). Cell lysates were analyzed by Western blotting using antibodies against FBXO38, KIF20B and actin.

While the role of FBXO38 in stabilizing KIF20B is not what is expected of an SCF complex, it is possible that an FBXO38-containing SCF complex may be affecting KIF20B indirectly by inducing degradation of a KIF20B destabilizing protein. Alternatively, FBXO38 might be acting independently of an SCF complex. We addressed these possibilities by comparing the effects of overexpression of FBXO38 on KIF20B levels to that of an FBXO38 mutant that lacks the F-box sequence needed to form the SCF complex. The F-box sequence in FBXO38 is located between amino acids 33 to 65^74^ and therefore we generated an F-box deletion mutant of FBXO38 (FBXO38ΔFBX) by truncating the first 77 amino acids. Co-expression of HA-KIF20B and either FLAG-FBXO38, FLAG-FBXO38ΔFBX or empty FLAG plasmid in AGS and 293T cells showed that both FBXO38 proteins greatly increased the levels of HA-KIF20B in both cell lines (Fig. 4A,B). Furthermore, immunoprecipitation of the FBXO38 proteins showed that FBXO38ΔFBX recovered similar amounts of KIF20B as WT FBXO38 but, unlike WT FBXO38, did not recover Skp1 (Fig. 4B). These results indicate that the F-box of FBXO38 (that is needed to form an SCF complex) is dispensable for KIF20B binding and stabilization, and therefore FBXO38 stabilizes KIF20B in an SCF-independent manner. In keeping with an SCF-independent effect of FBXO38, treatment of the cells with the MG132 proteasome inhibitor did not restore KIF20B levels caused by FBXO38 silencing (Fig. 4C).

### FBXO38 and USP7 regulate cytokinesis through KIF20B

**Figure 5:**
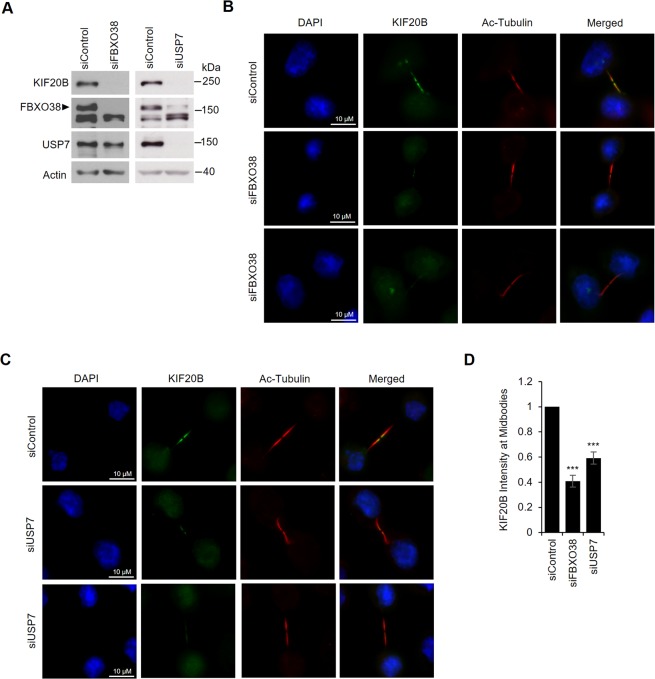
FBXO38 and USP7 silencing reduces KIF20B levels at the midbodies. AGS cells transfected with an siRNA targeting FBXO38, USP7 or a negative control siRNA were either lysed and analyzed by Western blotting using the indicated antibodies to confirm FBXO38 and USP7 silencing (**A**) or fixed and stained with DAPI and antibodies against KIF20B and acetylated tubulin (**B**,**C**). Representative fluorescence microscopy images of KIF20B localization at the midbodies after FBXO38 silencing (**B**) or USP7 silencing (**C**) are shown. (**D**) The fluorescence intensity of KIF20B at the midbodies in B and C was quantified for 30 midbodies in three independent experiments and values were normalized to the intensity of acetylated tubulin at the midbodies. Average values with standard deviations were plotted for FBXO38 and USP7 silencing shown relative to the silencing control. ***P < 0.001.

Since KIF20B’s function in cytokinesis involves its localization to midbodies^77,79,80^, we asked whether silencing FBXO38 and USP7 affected the level of KIF20B at midbodies. To this end, AGS cells treated with siRNA targeting FBXO38 or USP7 or negative control siRNA (Fig. 5A) were fixed and stained for KIF20B and acetylated tubulin to mark the midbody (Fig. 5B,C), and the level of KIF20B staining at the midbodies (relative to acetylated tubulin staining) was quantified for 30 midbodies per sample in three independent experiments (Fig. 5D). Silencing of either FBXO38 or USP7 resulted in a significant decrease in KIF20B at the midbodies, suggesting that FBXO38 and USP7 can affect KIF20B function in cytokinesis.

**Figure 6:**
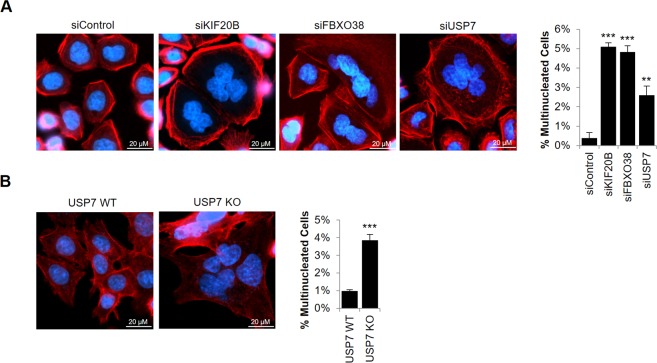
FBXO38 or USP7 depletion results in cytokinetic defects. (**A**) AGS cells were transfected with siRNA targeting KIF20B, FBXO38 or USP7 or with negative control siRNA (siControl). Cells were then fixed, stained with Phalloidin and DAPI, then imaged by fluorescence microscopy. The number of multinucleated cells in ~1500 cells were counted. Average values and standard deviation from three independent experiments are shown in the bar graph on the right. P values relative to siControl are indicated (* = 0.01 < *P* < 0.05; **= 0.001 < *P* < 0.01; ***P < 0.001). (**B**) HCT116 USP7 WT and USP7 KO cells were fixed and stained as in (A) and the number of multinucleated cells were determined. Average values and standard deviation from three independent experiments are shown in the bar graph on the right.

Previous reports have shown that KIF20B depletion results in the accumulation of multinucleated cells, due to defects in cytokinetic abscission^49,77,79,80^. To test whether USP7 and FBXO38 silencing affected KIF20B function in cytokinesis, we treated AGS cells with siRNA targeting each of the three proteins (see Fig. 3A,B,D for degree of silencing), or with a negative control siRNA and determined the number of multinucleated cells by microscopy of DAPI-stained cells. To differentiate multinucleated cells from overlapping single cells, we also stained for F-actin using fluorescently-labelled phalloidin to mark the outer edges of the cell. ~1500 cells in each sample were then counted and the percentage of cells containing two or more nuclei was determined. Sample images and quantification from three independent experiments are shown in Fig. 6A. Consistent with previous reports, KIF20B silencing resulted in a 13-fold increase in the frequency of multinucleated cells when compared to the silencing control. In addition, silencing of FBXO38 had almost an identical effect as silencing KIF20B on inducing multinucleated cells. USP7 silencing also obviously increased the percentage of multinucleated cells (~6-fold) relative to the silencing control. The results confirm that both FBXO38 and USP7 affect the cytokinetic function of KIF20B. The more modest effect of USP7 silencing (as compared to KIF20B and FBXO38 silencing) is consistent with the model that USP7 regulates KIF20B indirectly through FBXO38.

To further confirm the cytokinetic defect associated with USP7 we compared the number of multinucleated cells in HCT116 USP7 knockout cells or parental HCT116 cells. USP7 knockout cells exhibited a 4-fold higher frequency of multinucleated cells as the parental cells, in good agreement with the results of USP7 silencing in AGS cells (Fig. 6B).

**Figure 7:**
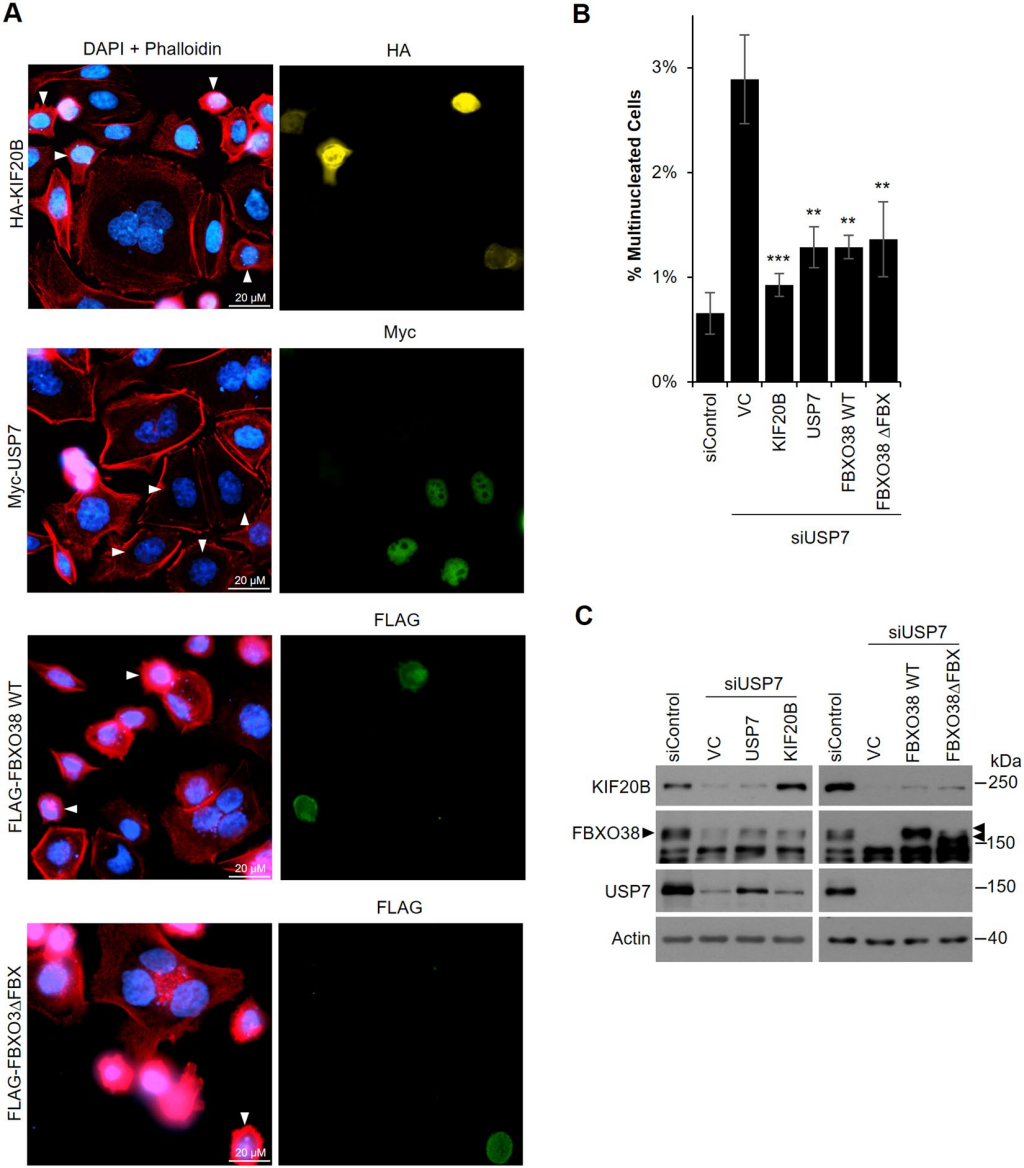
Overexpression of USP7, KIF20B and FBXO38 rescues the cytokinetic defect caused by USP7 silencing. (**A**) AGS cells were transfected with siRNA targeting USP7 followed by transfection with plasmids expressing HA-KIF20B, myc-USP7, FLAG-FBXO38 WT, FLAG-FBXO38∆FBX or an empty vector control (VC). Alternatively, cells were transfected with negative control siRNA (siControl) followed by transfection with the VC. Cells were then fixed, stained with DAPI and Phalloidin and with antibodies against either HA, myc or FLAG as indicated, then imaged by fluorescence microscopy. Cells expressing the tagged protein are indicated with white arrow heads in the left panel. The number of multinucleated cells (out of ~1500 cells/sample) in the silencing control or in USP7 silenced cells expressing the indicated tagged proteins or containing the VC were counted and plotted in (**B**). Average values from three independent experiments are shown along with standard deviations. P values are indicated for siUSP7 samples relative to siUSP7 with VC (* = 0.01 < *P* < 0.05; ** = 0.001 < *P* < 0.01; ***P < 0.001). (**C**) Whole cell lysates of cells treated in (A) were analyzed by Western Blotting using the indicated antibodies.

Since USP7 is known to regulate the levels of multiple proteins, it is possible that the multinucleated phenotype that we found to be associated with USP7 depletion is independent of FBXO38 and KIF20B. For example, USP7 has been reported to affect Aurora A, whose loss of function is associated with a multipolar spindle phenotype that can lead to multinucleated cells^49,87,88^. We examined the possibility that USP7 silencing in AGS cells increased the incidence of multipolar spindles but found no cells with multipolar spindles in 50 metaphase cells examined (Supplementary Fig. S4). In addition, we tested whether the multinucleated phenotype associated with USP7 silencing was caused by loss of FBXO38 and KIF20B by performing rescue experiments. To this end, AGS cells were treated with siRNA targeting the 3′UTR of USP7 or a negative control siRNA, then transfected with a plasmid expressing myc-USP7, HA-KIF20B, FLAG-FBXO38 or an empty plasmid. Cells were then stained with antibodies against FLAG, HA or myc to identify cells expressing the transfected plasmid and also stained with DAPI and fluorescently-labelled phalloidin to identify multinucleated cells (Fig. 7A,B). As expected, USP7 silencing (which was confirmed by Western blotting; Fig. 7C) in the presence of the control plasmid resulted in an increase in multinucleated cells when compared to the silencing control (Fig. 7B). Also as expected, this phenotype was greatly reduced (compared to the empty vector control) in cells in which myc-USP7 was expressed. In addition, we found that USP7 silenced cells expressing HA-KIF20B or FLAG-FBXO38 had greatly reduced numbers of multinucleated cells, with HA-KIF20B almost completely rescuing the phenotype. Note that since only a small percentage of the USP7-silenced cells express the recombinant proteins, only a small effect on FBXO38 levels upon USP7 rescue and on KIF20B levels upon USP7 and FBXO38 rescue can be seen by western blots of total cells (Fig. 7C). The data strongly suggest that the cytokinetic defect observed upon USP7 silencing is a result of decreased KIF20B levels. The same rescue experiments were also performed with FBXO38ΔFBX, giving almost identical results to WT FBXO38 and further confirming that FBXO38 is functioning independently from an SCF complex in its regulation of KIF20B levels and function. Together the results indicate that USP7 and FBXO38 can affect cytokinesis through effects on KIF20B.

## Discussion

USP7 is known to bind and deubiquitylate multiple proteins with diverse functions, thereby regulating a variety of cellular pathways relevant for oncogenesis. To gain additional insight into the protein interactions and functions of USP7, we used an AP-MS proteomics approach to identify USP7 binding partners in the context of gastric carcinoma cells. This led to the identification of a previously uncharacterized interaction with FBXO38. We now show that the USP7-FBXO38 interaction has a role in regulating cytokinesis through KIF20B.

We have shown that FBXO38 interacts with USP7, predominantly through the USP7 NTD binding pocket, that is also known to bind p53, Hdm2, HdmX, MCM-BP, Epstein-Barr virus EBNA1 and Kaposi’s sarcoma associated herpesvirus (KSHV) vIRF1 and vIRF4^24,26,33,55,59–61^. We have previously defined a P/A/ExxS motif that is used to bind this NTD binding pocket^26,59,61^ and FBXO38 has 8 such motifs that might be responsible for this interaction. We also observed that mutation of the USP7 Ubl2 binding pocket consistently decreased FBXO38 binding, suggesting that part of FBXO38 might also contact this pocket. Other proteins that bind the Ubl2 pocket do so using a polybasic KxxxK motif^62^, and FBXO38 contains five of these motifs that may contribute to USP7 binding.

The interaction of USP7 with FBXO38 prompted us to examine a role of USP7 in stabilizing FBXO38 through deubiquitylation. In support of this hypothesis, we found that FBXO38 levels increased upon overexpression of WT USP7 but not the USP7 catalytic mutant (Fig. 1C) as well as upon treatment with the MG132 protease inhibitor (Fig. 4C). Note that this effect on FBXO38 levels is more readily detected when FBXO38 is co-overexpressed with USP7 than on endogenous FBXO38, because the transfected USP7 is only expressed in a minority of the cells. Furthermore, depletion of USP7 in several cell lines dramatically decreased endogenous levels of FBXO38, an effect that could be reversed upon inhibition of the proteasome (Fig. 1D). Finally inhibition of the catalytic activity of USP7 also decreased FBXO38. Together these findings suggest that FBXO38 is an unstable protein that is stabilized by USP7 through deubiquitylation, preventing its proteasomal degradation. However, it is also possible that USP7 affects FBXO38 stability indirectly through deubiquitylation of another protein that stabilizes FBXO38.

FBXO38 has not been extensively studied but has been found to be a coactivator of the KLF7 transcription factor, which is a regulator of neuronal differentiation^74–76^. FBXO38 (also called MoKA) was shown to bind KLF7 through its F-box domain and to enhance transcription of KLF7 target genes, including p21^Waf/Cip^^74^. Furthermore, a dominant mutation in FBXO38 that renders it unable to bind KLF7 causes spinal muscular atrophy (SMA), an inherited disorder characterized by progressive muscle weakness^75^. FBXO38 has also been found to shuttle between the nucleus and the cytoplasm, suggesting that it also has cytoplasmic functions^74,76^. To gain a more comprehensive profile of the protein interactions of FBXO38 that might reflect additional functions, we performed a BioID experiment with FBXO38. This revealed several novel interactions including one with KIF20B. In addition, the SCF complex components, SKP1 and CUL1, were prominent interactors showing that FBXO38, like other F-box proteins, is capable of forming an SCF ubiquitin ligase complex. This suggests that FBXO38 may have roles in the ubiquitylation and degradation of specific target proteins, which may include some of the proteins identified as FBXO38 interactors in the BioID experiment. However, FBXO38 was found to stabilize KIF20B and this stabilization did not require the F-Box motif necessary to form the SCF complex. Therefore FBXO38 stabilizes KIF20B independently from the SCF complex. Together, our results support a model where USP7 binds and stabilizes FBXO38, which in turn, binds and stabilizes KIF20B.

Our finding that FBXO38 acts independently of an SCF complex to stabilize KIF20B, is consistent with the KLF7 co-activator function of FBXO38, which was also shown to be independent of an SCF complex^74^. Furthermore, SCF-independent functions have been reported for ~12% of human F-box proteins^89^. This includes FBXO7, which has both SCF-dependent and independent functions, and resembles FBXO38 in that it binds and stabilizes specific proteins (p27 and Cdk6 complexes)^89–91^. Although there are currently no known SCF-dependant functions of FBXO38, the fact that we showed it could interact with SKP1 and CUL1 suggests that, like FBXO7, it might have additional SCF-dependant functions. While FBXO38 functions with KLF7 and KIF20B are both SCF-independent, the mechanisms of these interactions are distinct; FBXO38 binds KLF7 through its F-box sequence^74^, while FBXO38 lacking this sequence binds KIF20B, indicating that the KIF20B binding site is elsewhere in the protein. FBXO38 contains a central RNase Inhibitor (RNI)-like domain, similar to that of leucine-rich repeats (LRRs) in placental RNase inhibitor^92^. LRRs are protein interaction domains^93,94^ and therefore this domain might mediate the KIF20B interaction.

KIF20B (previously called MPHOSPH1 or MPP1) is one of three members of the Kinesin-6 family and is a critical regulator of cytokinesis^77–80,95^. In cytokinesis a contractile ring drives the ingression of a cleavage furrow that partitions the cytoplasm between the daughter cells that are connected through an intercellular bridge. A midbody forms at the center of the intercellular bridge and serves as both the anchor for the ingressed cleavage furrow and a platform for the assembly of the machinery that drives abscission. KIF20B has been shown to promote cytokinesis by temporally regulating both cytokinetic furrow ingression and abscission and also by promoting efficient midbody maturation and microtubule stabilization^80^. In keeping with these important roles, multiple studies have shown that knockdown of KIF20B in bladder, liver and HCT116 colon cancer cells results in cytokinetic defects that are reflected in accumulation of multinucleated cells that eventually undergo apoptosis^33,57,79,80^. The major effect of FBXO38 and USP7 on KIF20B levels, prompted us to investigate whether these two proteins are also important for cytokinesis. As predicted, silencing of USP7 or FBXO38 or USP7 knockout significantly increased the percentage of multinucleated cells. Furthermore, rescue experiments showed that multinucleated cells caused by lack of USP7 could be prevented by providing KIF20B or FBXO38, confirming that cytokinetic defects associated with USP7 depletion are a result of decreased FBXO38 and KIF20B levels. The decreased levels of KIF20B observed at the midbodies upon depletion of FBXO38 or USP7 further supports the contention that these proteins are affecting cytokinesis through disruption of KIF20B function.

In conclusion, we have identified novel roles for USP7 and FBXO38 in cytokinesis through the stabilization of KIF20B. While USP7 has several identified roles in cell cycle progression^13,14,48,49,55,56,58^, to our knowledge, this is the first report of a role for USP7 in cytokinesis. Overexpression of KIF20B has been found to positively correlate with tumorigenesis and cell proliferation in bladder and liver cancer^79,95^. Consequently, KIF20B is recognized as a potential therapeutic target for these cancers, since depletion of KIF20B in these cells leads to mitotic arrest, through failure of cytokinesis, and subsequent apoptosis. Similarly, the overexpression of USP7 that has been reported in many cancer cells may also induce their proliferation by upregulating KIF20B and promoting cytokinesis. Our findings suggest that USP7 and FBXO38 may both be useful targets for inhibiting the growth of cancer cells with KIF20B overexpression.

## Materials and Methods

### Cell Lines

AGS human gastric adenocarcinoma cells were maintained in in RPMI 1640 supplemented with 10% fetal bovine serum (FBS, Wisent Inc). HEK293T and HEK293A human embryonic kidney cells were maintained in Dulbecco’s modified Eagle medium (DMEM) supplemented with 10% FBS. The HCT116 human colon carcinoma cells (received from Daniel Durocher) and USP7-null HCT116 cells (obtained from Bert Vogelstein) were maintained in minimal essential medium (MEM alpha) supplemented with 10% FBS. All media was purchased from Life Technologies Inc.

### Plasmids and siRNA

Plasmids expressing myc-USP7 (pCDNA3), the myc-USP7 catalytic mutant (C223S) and myc-USP7 D762R/D764R (UBL2) were described previously^47,62^. Myc-USP7 (DW) and myc-USP7 D164A/W165A/D762R/W764A (DW/Ubl2) were gifts from Yi Sheng and Vivian Saridakis, respectively^96^. pME-HA-KIF20B and PCDNA6:FLAG-FBXO38 were gifts from Masatoshi Hagiwara and Charlotte Sumner, respectively and were described previously^75,97^. The FLAG-FBXO38 mutant lacking the first 77 amino acids that contains the F-Box domain (FLAG-FBXO38∆FBX) was generated by PCR amplification of the FBXO38 sequence in PCDNA6:FLAG-FBXO38 using forward primer: 5′-CATGGCGATCGCACCATG GCAGGGCGCTGGTGGGAATATATG-3′ and reverse primer: 5′-CATGACGCGTAATGTAGTCGTCTTCAACTGGCTC-3′. The resulting PCR product was inserted between the Sgf1 and Mlu1 sites of the original FLAG-FBXO38 pcDNA6 vector. The FBXO38 WT and ∆FBX plasmids were verified by DNA sequencing. Stealth siRNA targeting FBXO38 (#1 5′-GGUGGUGGCCGAGAGUGGAAAUAAU-3′ and #2 5′-CCACAGCCAUUUAAAGACUUCCUUU-3′) and USP7 (#1 5′-CCCAAATTATTCCGCGGCAAA-3′ and #2 5′-CCTCTAGCCGAAGTCTTCAGCAAGA-3′) were from Invitrogen. A previously characterized siRNA targeting KIF20B (GTGAAGAAGTGCGACCGAA^79^) was synthesized by SIGMA. AllStar negative-control siRNA was obtained from Qiagen.

### Transfections and USP7 Inhibitor Treatment

Approximately 6 × 10^5^ AGS or HCT116 cells in 10-cm dishes or on coverslips (for microscopy) were transfected with 100 pmol of small interfering RNA (siRNA) against USP7, FBXO38 or KIF20B using 4 μl of Lipofectamine 2000. For USP7 and FBXO38 silencing, siRNA transfections were repeated two additional times after 24 and 48 h. Cells were harvested 48 h after the last round of transfection and processed for Western blotting, microscopy or mRNA quantification as described below. For proteasome inhibition, 10 µM final concentration of MG132 (Sigma) was added to the cells 12 hours before harvesting. For overexpression experiments, 293 T or AGS cells at ~80% confluency in 10-cm dishes were transfected with 7 µg of the indicated plasmids using transfection grade Polyethylenimine (PEI, Polyscience 23966). In brief, 7 µg DNA was diluted in 1 ml optimum media, followed by addition of 21 µl PEI reagent. The DNA-PEI complexes were mixed briefly by vortexing and incubated at room temperature for 15 minutes prior adding to the cells. Cells were harvested 48 hours post-transfection. For the rescue experiments after USP7 silencing, 7 hours after the last round of USP7 siRNA transfection, AGS cells were transiently transfected with plasmids expressing myc-USP7, HA-KIF20B and FLAG-FBXO38 or FLAG-FBXO38∆FBX as described above, then harvested 48 hours post-transfection and processed for microscopy or Western blotting. For the USP7 inhibitor experiments, AGS and HCT116 cells at ~40% confluency were treated with a final concentration of 5 µM of USP7 inhibitor (Compound 4; also known as AD04) or the inactive enantiomer (gifts from Timothy Harrison^15^). Cells were harvested or 24 hours post-inhibitor treatment.

### Generation of Recombinant Adenovirus

To generate adenovirus expressing USP7 or LacZ (negative control) with C-terminal sequential peptide affinity (SPA)-tag^98^, the coding sequence for the SPA tag was first inserted between the EcoRI and NotI sites of the multicloning site of pENTR4 (Life Technologies). The USP7 or LacZ coding sequences were then individually inserted between the BamHI and KpnI sites of the multicloning sites to generate constructs expressing C-terminally tagged USP7 or β-Gal. USP7-SPA and LacZ-SPA were transferred to the pAd⁄CMV⁄V5-DEST adenovirus expression vector using the ViraPower™ Adenoviral Gateway™ expression kit (Invitrogen). Adenovirus was generated and amplified in HEK293A cells as per the manufacturer’s instructions. The virus was titred onto AGS cells, followed by immunofluorescence microscopy and Western blotting using antibody against the FLAG epitope in the SPA tag (F1804 from Sigma; 1:800 dilution) to verify expression of the full length proteins and determine the minimum amount of virus that could be used to infect most of the cells.

### Affinity Purification Coupled to Mass Spectrometry (AP-MS)

AGS cells in 25 15-cm diameter plates at 70% confluence were transduced with the adenoviruses expressing USP7 or LacZ (minimum amount required to infect 70% of the cells) and harvested 48 hours post-transduction. Cells were lysed and extracted by the “high salt extraction” method that we have previously described^99^. In brief, cells were resuspended in 1.33 pellet volumes of TpA (10 mM HEPES pH 7.9, 1.5 mM MgCl_2_, 10 mM KCl, 0.5 mM DTT and P8340 protease inhibitor) followed by 10 strokes in a dounce homogenizer. 1 pellet volume of TpB (50 mM HEPES pH 7.9, 1.5 mM MgCl_2_, 0.5 mM DTT, 1.26 M potassium acetate and 75% glycerol) was added to the lysate followed by 10 additional strokes in the homogenizer. After incubation on ice for 30 minutes, the extract was clarified by centrifugation and subsequently dialyzed against 10 mM HEPES pH 7.9, 0.1 M potassium acetate, 0.1 mM EDTA, 0.1 mM DTT, 10% glycerol. After dialysis, the extract was incubated with 50 μl of anti-FLAG M2 resin (Sigma-Aldrich) 4 hours at 4 °C with end-over-end rotation. The resin was washed first in IPP buffer (10 mM Hepes pH 7], 100 mM NaCl, 0.1% Triton, 10% glycerol) followed by a second wash in 50 mM ammonium bicarbonate, 75 mM KCl. Immunoprecipitated proteins were eluted three times in 150 μl 0.5 M ammonium hydroxide, pH 11. Eluates were dried by lyophilisation using a SpeedVac, washed once in high-performance liquid chromatography (HPLC)-grade water and further dried by lyophilisation. The lyophilized protein was resuspended in 50 mM ammonium bicarbonate containing 10 µg/µl proteomic grade trypsin (Sigma) and incubated overnight at 37 °C followed by further incubation for 2 hours in freshly added trypsin. Samples were lyophilised again and the peptides were analyzed by liquid chromatography-tandem mass spectrometry (LC-MS/MS) using a LTQ Orbitrap system (Thermo Finnigan) and identified using Mascot software (Matrix Science, United Kingdom).

### Identification of FBXO38 Interactions by BioID

BioID^100^ was carried out essentially as we described previously^101^. In brief, full length FBXO38 (BC050424) coding sequence was amplified by PCR, and cloned into our pcDNA5 FRT/TO FLAGBirA* expression vector. Using the Flp-In system (Invitrogen), HEK293TREx Flp-In cells (Thermo Fisher Scientific) stably expressing FLAGBirA* alone or FLAGBirA*–FBXO38 were generated. After selection (DMEM + 10% FBS + 200 μg/ml hygromycin B), two independent replicates of five 150 cm^2^ plates of sub-confluent (60%) cells were incubated for 24 hrs in complete media supplemented with 1 µg/ml tetracycline (Sigma), 50 µM biotin (BioShop). Cells were collected and pelleted (2,000 rpm, 3 min), the pellet was washed twice with PBS, and dried pellets were snap frozen. The cell pellet was resuspended in 10 ml of lysis buffer (50 mM Tris-HCl pH 7.5, 150 mM NaCl, 1 mM EDTA, 1 mM EGTA, 1% Triton X-100, 0.1% SDS, 1:500 protease inhibitor cocktail (Sigma-Aldrich), 1:1000 benzonase nuclease (Novagen) and incubated on an end-over-end rotator at 4 °C for 1 hour, briefly sonicated to disrupt any visible aggregates, then centrifuged at 16,000 g for 30 min at 4 °C. Supernatant was transferred to a fresh 15 mL conical tube. 30 μl of packed, pre-equilibrated Streptavidin sepharose beads (GE) were added and the mixture incubated for 3 hours at 4 °C with end-over-end rotation. Beads were pelleted by centrifugation at 2000 rpm for 2 min and transferred with 1 mL of lysis buffer to a fresh Eppendorf tube. Beads were washed once with 1 mL lysis buffer and twice with 1 mL of 50 mM ammonium bicarbonate (pH = 8.3). Beads were transferred in ammonium bicarbonate to a fresh centrifuge tube, and washed two more times with 1 ml ammonium bicarbonate buffer. Tryptic digestion was performed by incubating the beads with 1 µg MS-grade TPCK trypsin (Promega, Madison, WI) dissolved in 200 μl of 50 mM ammonium bicarbonate (pH 8.3) overnight at 37 °C. The following morning, 0.5 μg MS-grade TPCK trypsin was added, and beads were incubated 2 additional hours at 37 °C. Beads were pelleted by centrifugation at 2000 × g for 2 min, and the supernatant was transferred to a fresh Eppendorf tube. Beads were washed twice with 150 µL of 50 mM ammonium bicarbonate, and these washes were pooled with the first eluate. The sample was lyophilized, and resuspended in buffer A (0.1% formic acid). 1/5th of the sample was analyzed per MS run.

High performance liquid chromatography was conducted using a 2 cm pre-column (Acclaim PepMap 50 mm × 100 um inner diameter (ID)), and 50 cm analytical column (Acclaim PepMap, 500 mm × 75 um diameter; C18; 2 um; 100 Å, Thermo Fisher Scientific, Waltham, MA), running a 120 min reversed-phase buffer gradient at 225 nl/min on a Proxeon EASY-nLC 1000 pump in-line with a Thermo Q-Exactive HF quadrupole-Orbitrap mass spectrometer. A parent ion scan was performed using a resolving power of 60,000, then up to the twenty most intense peaks were selected for MS/MS (minimum ion count of 1,000 for activation) using higher energy collision induced dissociation (HCD) fragmentation. Dynamic exclusion was activated such that MS/MS of the same *m/z* (within a range of 10 ppm; exclusion list size = 500) detected twice within 5 sec were excluded from analysis for 15 sec. For protein identification, Thermo. RAW files were converted to the mzXML format using Proteowizard^102^, then searched using X!Tandem^103^ and Comet^104^ against the human Human RefSeq Version 45 database (containing 36,113 entries). Search parameters specified a parent ion mass tolerance of 10 ppm, and an MS/MS fragment ion tolerance of 0.4 Da, with up to 2 missed cleavages allowed for trypsin. Variable modifications of +16@M and W, +32@M and W, +42@N-terminus, and +1@N and Q were allowed. Proteins identified with an iProphet cut-off of 0.9 (corresponding to ≤1% FDR) and at least two unique peptides were analyzed with SAINT Express v.3.6. Sixteen control runs (from cells expressing the FlagBirA* epitope tag) were collapsed to the two highest spectral counts for each prey, and compared to the two technical of each of the two biological replicates of FBXO38 BioID. High confidence interactors were defined as those with BFDR ≤ 0.01.

### Immunofluorescence Microscopy

For identification of multinucleated cells, cells transfected with siRNA targeting KIF20B, USP7 or FBXO38 or the silencing control were grown on coverslips and fixed with 3.7% formaldehyde in phosphate-buffered saline (PBS) for 20 min, rinsed once in PBS and permeabilized with 1% Triton X-100 in PBS for 5 min. Samples were blocked with 4% bovine serum albumin (BSA) in PBS followed by incubation with Phalloidin Alexa Fluor 647 (Invitrogen A22287; 1:1000 dilution) in 4% BSA. Coverslips were mounted on slides using ProLong Gold antifade medium with DAPI (4′,6-diamidino-2-phenylindole) (Invitrogen). Images were obtained using the 40x oil objective on a Leica inverted fluorescence microscope and processed using the Leica Application Suite X (LAS X, version 3.3.0) software program. ~1500 cells per sample were examined for the number of multinucleated cells (cells containing two or more nuclei) in three independent experiments. Averages and standard deviations were calculated in Excel. *P* values were determined using two-tailed *t*-tests in Excel. For the multinuclei rescue experiment, cells transfected with siRNA targeting USP7 or the silencing control were fixed, permeabilized and blocked as described above, followed by incubation with primary antibodies against HA (Santa Cruz 805; 1:500 dilution), FLAG (F1804 from Sigma; 1:800 dilution) and myc (Santa Cruz 789; 1:500 dilution) in 4% BSA. After washing in PBS, cells were incubated with Phalloidin Alexa Fluor 647 (Invitrogen A22287; 1:1000 dilution) and secondary antibodies goat anti-rabbit or goat anti-mouse Alexa Fluor 488 (Invitrogen; 1:800 dilution) in 4% BSA. Coverslips were mounted and images were obtained and processed as described above. For quantification of KIF20B flourescence intensity at the midbodies, cells silenced for FBXO38 or USP7 or transfected with the silencing control were fixed in 100% methanol for 15 minutes at −20 °C. After blocking, cells were incubated with antibodies against KIF20B (Santa Cruz 515194; 1:50) and acetylated tubulin (Santa Cruz 82557; 1:800). After washing in PBS, cells were incubated with secondary antibodies goat-anti mouse IgG_1_ Alexa Fluor 488 and goat-anti mouse IgG_2b_ Alexa Fluor 594 (Invitrogen; 1:800 dilution) in 4% BSA. Coverslips were mounted and images were obtained and processed as described above using a 60x oil objective. Exposure times used were the same for all samples and images were taken on the same day. The fluorescence intensity of KIF20B was quantified using Fiji/ImageJ (http://imagej.net/Fiji/Downloads) for 30 midbodies per sample for each of three independent experiments, and values were normalized to the acetylated tubulin staining at the midbodies.

### Western Blotting

Cells were lysed in 9 M urea, 10 mM Tris-HCl pH 6.8, sonicated and clarified by centrifugation. 80 µg of protein was subjected to SDS-PAGE and transferred to nitrocellulose. Membranes were blocked in 5% non-fat dry milk in TBS-T (TBS with 0.1% Tween), then incubated with primary antibodies: mouse FLAG M2 (F1804 from Sigma; 1:5000 dilution), rabbit anti-FLAG (PA1-984B from Invitrogen; 1:5000 dilution), rabbit anti-HA (Santa Cruz 805; 1:200 dilution), rabbit anti-myc (Santa Cruz 789; 1:5000 dilution), rabbit anti-USP7 (Bethyl laboratories A300-033A; 1:10000 dilution), mouse anti-KIF20B (Santa Cruz 515194; 1:200 dilution), rabbit anti-FBXO38 (Bethyl laboratories A302-378A; 1:5000 dilution), rabbit anti-SKP1 (Santa Cruz 7163; 1:200 dilution), goat anti-actin (Santa Cruz 1615; 1:1000 dilution). Membranes were washed three times in TBS-T, followed by incubation with goat anti-mouse HRP (Santa Cruz 2005), goat anti-rabbit HRP (Sigma SAB3700878) or donkey anti-goat HRP (Sigma SAB3700285) at 1:5000 dilution. Membranes were developed using chemiluminescence reagents (ECL or ECL-prime; Santa Cruz or Amersham). Protein bands were quantified using ImageQuantTL (GE Healthcare Sciences) and normalized to actin bands.

### RNA Extraction and KIF20B mRNA Quantification

Total RNA was isolated from cells transfected with the silencing control or FBXO38 siRNA (as described above) using TRIzol (Invitrogen). The quantity and quality of the extracted RNA were determined by reading the optical densities at 260 and 280 nm using a NanoDrop spectrophotometer (Thermo Scientific). To quantify the KIF20B or actin (control) mRNA, 1 μg of total RNA was reverse transcribed in a 20 μl reaction mixture using SuperScript III reverse transcriptase (Life Technologies) and random hexamer primers according to the manufacturer’s instructions. Quantitative real-time PCR was performed using 0.2 μl of the cDNA and SsoFast EvaGreen Supermix (Bio-Rad) on the CFX384 Touch Real Time PCR Detection System (Bio-Rad). Primers used were: KIF20B forward 5′-AGA AAC CAA CAG GCA AGA AAC-3′, KIF20B reverse 5′-CTC ATC ACG CAT TAC AGA TAC C-3′, actin forward 5′-GGACTTCGAGCAAGAGATGG-3′ and actin reverse 5′-AGCACTGTGTTGGCGTACAG-3′. Experiments were performed in triplicate and KIF20B mRNA signals in each sample was normalized to the actin mRNA signal.

### Immunoprecipitation

Cells co-transfected with the FLAG-FBXO38 and the myc-USP7 expression constructs or the myc-USP7 construct alone were lysed on ice for 30 min in 4X volume of radioimmunoprecipitation assay (RIPA) buffer (50 mM Tris-HCl pH 8, 150 mM NaCl, 0.1% sodium deoxycholate, 0.5% NP-40) with complete protease inhibitors (Sigma P8340), followed by sonication and clarification by centrifugation. For the co-expression experiments, 2 mg of each clarified lysate was incubated with 10 μl of M2 anti-FLAG resin (Sigma) overnight at 4 °C with end-over-end rotation. Cells with myc-USP7 constructs alone were lysed in RIPA buffer and incubated with 10 μl of anti-Myc resin (c-myc AC – sc-40 AC Santa Cruz) overnight at 4 °C with end-over-end rotation. For immunoprecipitation of FLAG-FBXO38 with HA-KIF20B or endogenous KIF20B, cells were lysed in 4X volume of lysis buffer (50 mM Tris-HCl pH 8.0, 150 mM NaCl, 10% glycerol and 0.1% NP-40). FLAG-tagged FBXO38 was immunoprecipitated using anti-FLAG M2 resin as described above. All resins were harvested by centrifugation, washed 4 times in lysis buffer, then boiled in 2x SDS loading buffer. Recovered proteins were separated by SDS-PAGE and analyzed by Western blotting, as described above.

## Supplementary information

Supplementary Figures

Table S1

Table S2

Table S3
