# Supplementary material for: USP7 Regulates Cytokinesis through FBXO38 and KIF20B
Source: Sci Rep. 2019 Feb 25;9:2724. doi: 10.1038/s41598-019-39368-y (PMC6389929; doi:10.1038/s41598-019-39368-y)
Supplement: Supplementary file 1 — Supplementary Figures [file 41598_2019_39368_MOESM1_ESM.pdf]

USP7 Regulates Cytokinesis through FBXO38 and KIF20B

Anna Georges, Etienne Coyaud, Edyta Marcon, Jack Greenblatt, Brian Raught and Lori Frappier

Supplementary Figures

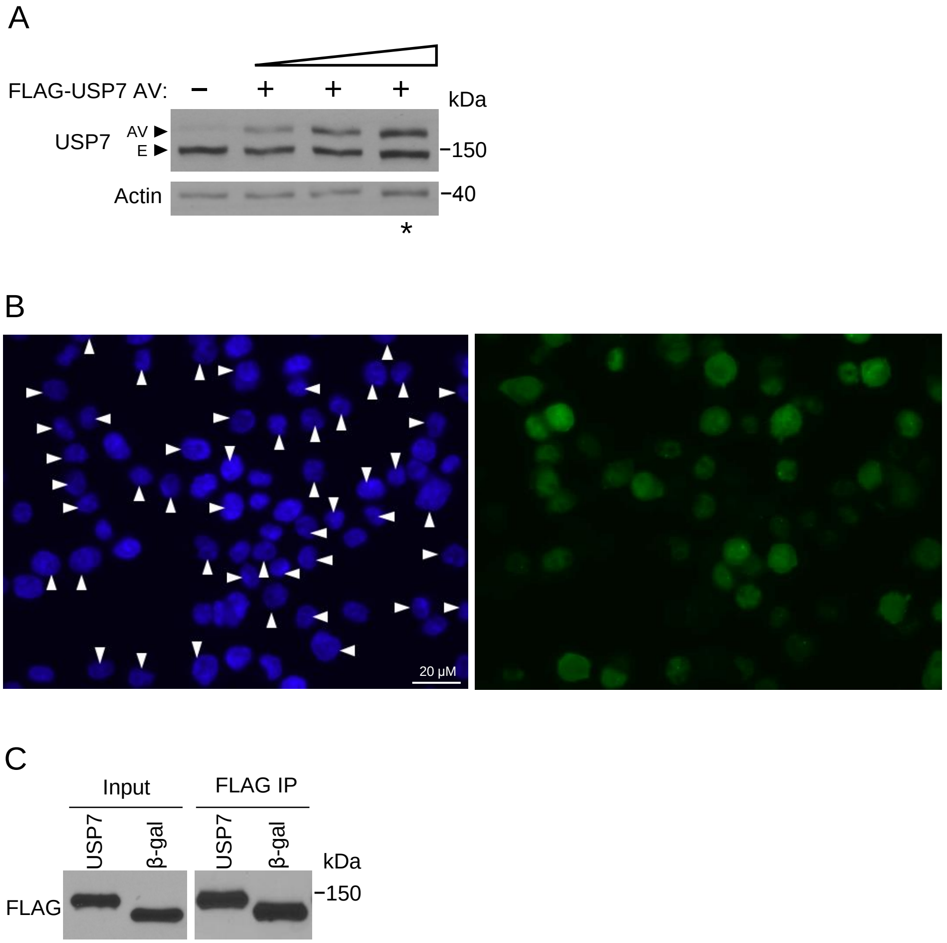

Supplementary Figure S1. Expression of FLAG-USP7 delivered using an adenovirus expression system.
(A) Adenovirus expressing FLAG-tagged USP7 (FLAG-USP7 AV) was titred onto AGS cells. Cells were then harvested 48 hours post-transduction and analyzed by Western blotting using an antibody against USP7 to compare the levels of FLAG-USP7 (AV) to endogenous USP7 (E). The viral titre used for the AP-MS experiment is indicated by a * under the gel. (B) AGS cells were transduced with adenovirus expressing FLAG-tagged USP7 at the viral titre indicated in (A). Cells harvested 48 hours post-transduction were fixed and stained for FLAG (right) and counter stained with DAPI (left) then imaged by microscopy. DAPI stained cells expressing FLAG-USP7 are indicated with white arrow heads. (C) AGS cells transduced with adenoviruses expressing FLAG-USP7 (as in B) or FLAG- β-gal were lysed and processed as described in methods for AP-MS. FLAG-proteins were recovered on and eluted from anti-FLAG resin. 10% of the starting lysate (input) and FLAG resin elution (FLAG eluate) were analyzed by Western blotting using anti- FLAG antibody. The remainder of the elution was processed for LC-MS/MS and gave the results in Table 1.

Supplementary Figure S2. Uncropped Western blots

Figure 1A

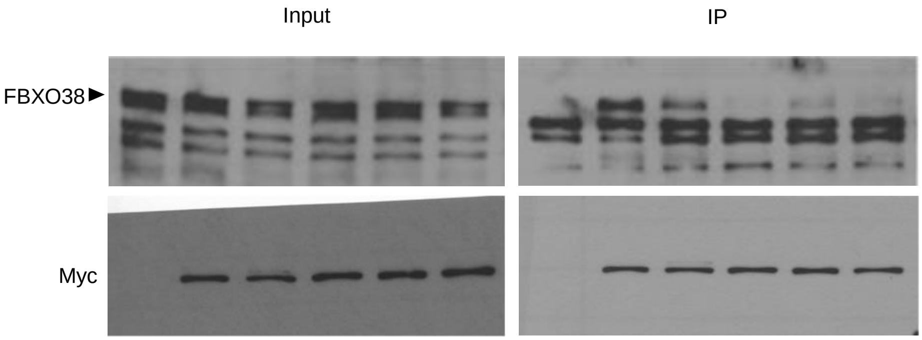

Figure 1B

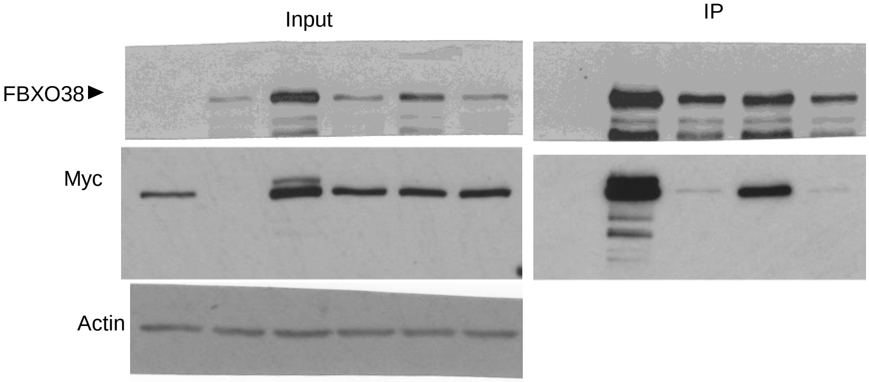

Figure 1C

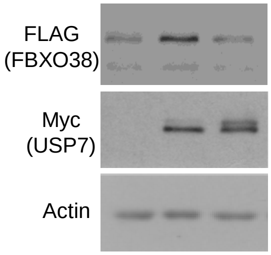

Figure 1D

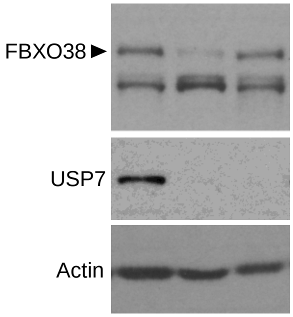

Figure 2B

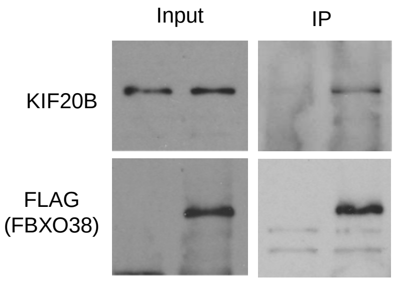

Figure 3A

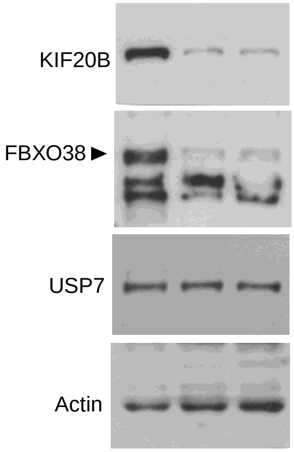

Figure 3B

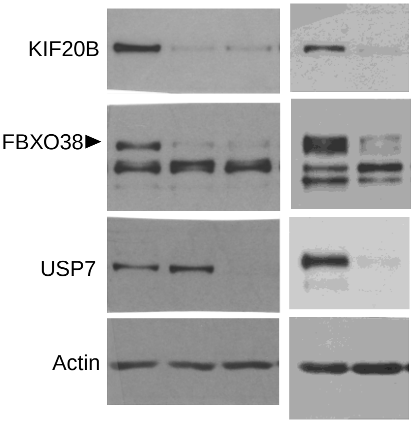

Figure 3E

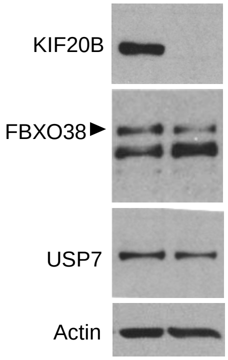

Figure 3F

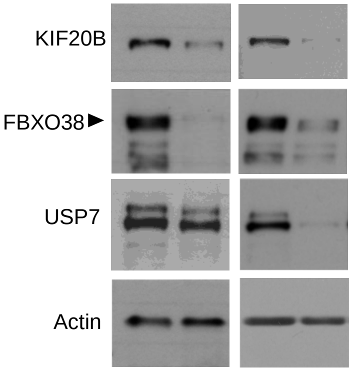

Figure 3G

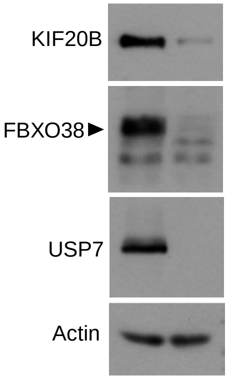

Figure 3H

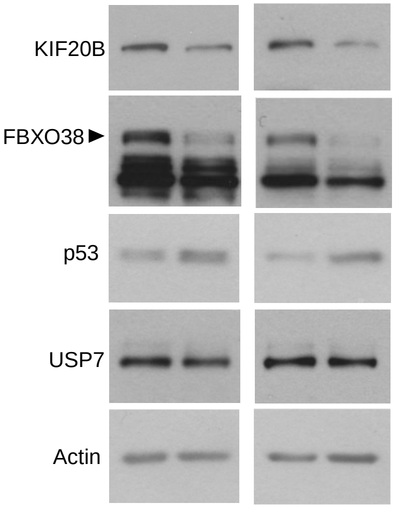

Figure 4A

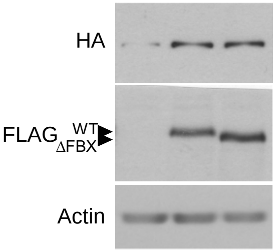

Figure 4B

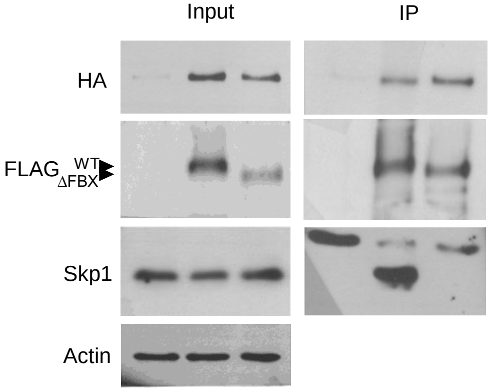

Figure 4C

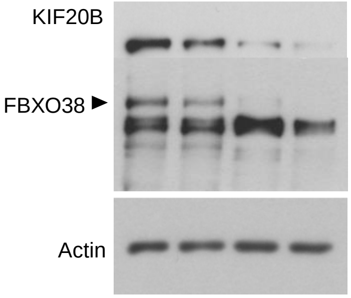

Figure 5A

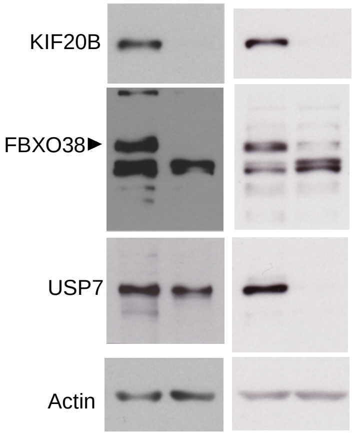

Figure 7C

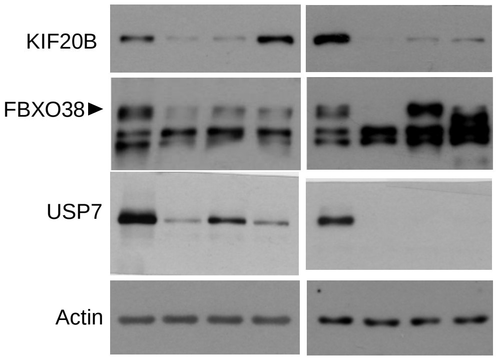

Figure S1A

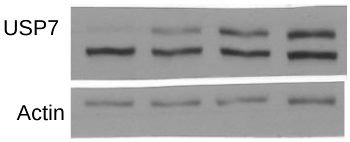

Figure S1C

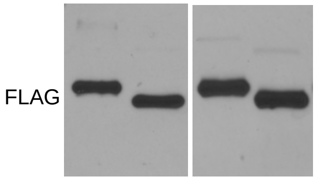

Figure S3A

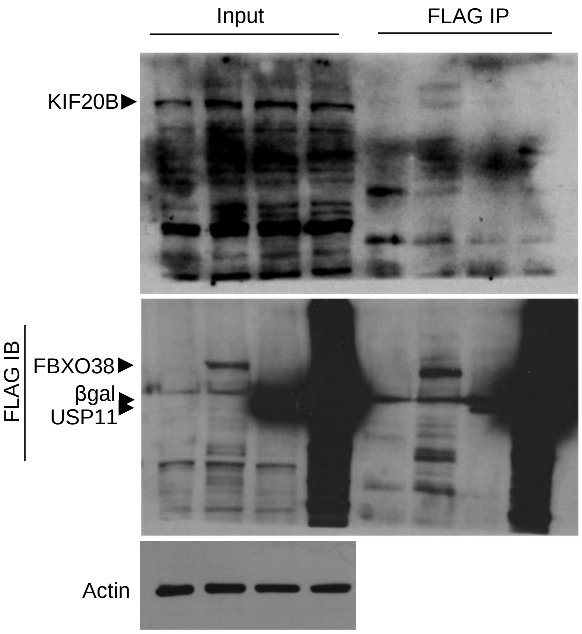

Figure S3B

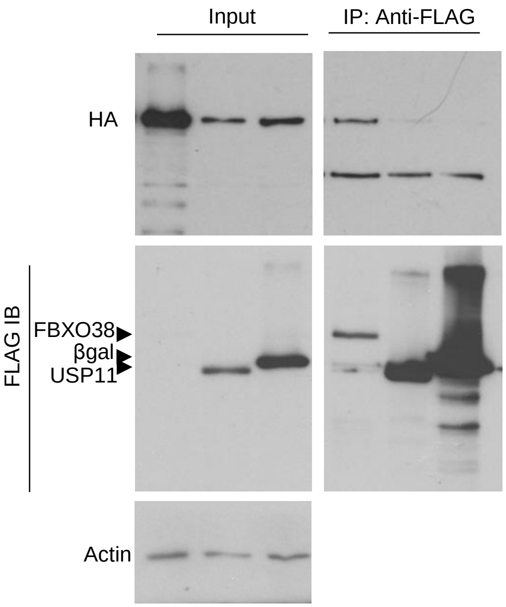

Figure S4C

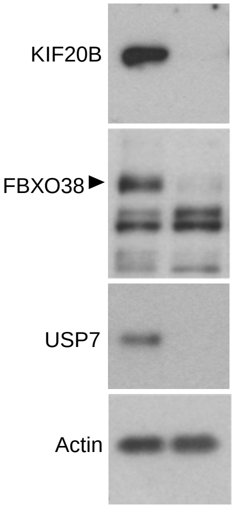

Supplementary Figure S2. Uncropped Western blots. Uncropped Western blots for each figure in the manuscript are shown as indicated. Note that the membranes themselves were cut into sections to enable probing of the same blots with multiple antibodies. Therefore the above membrane sections are the full blots for each antibody.

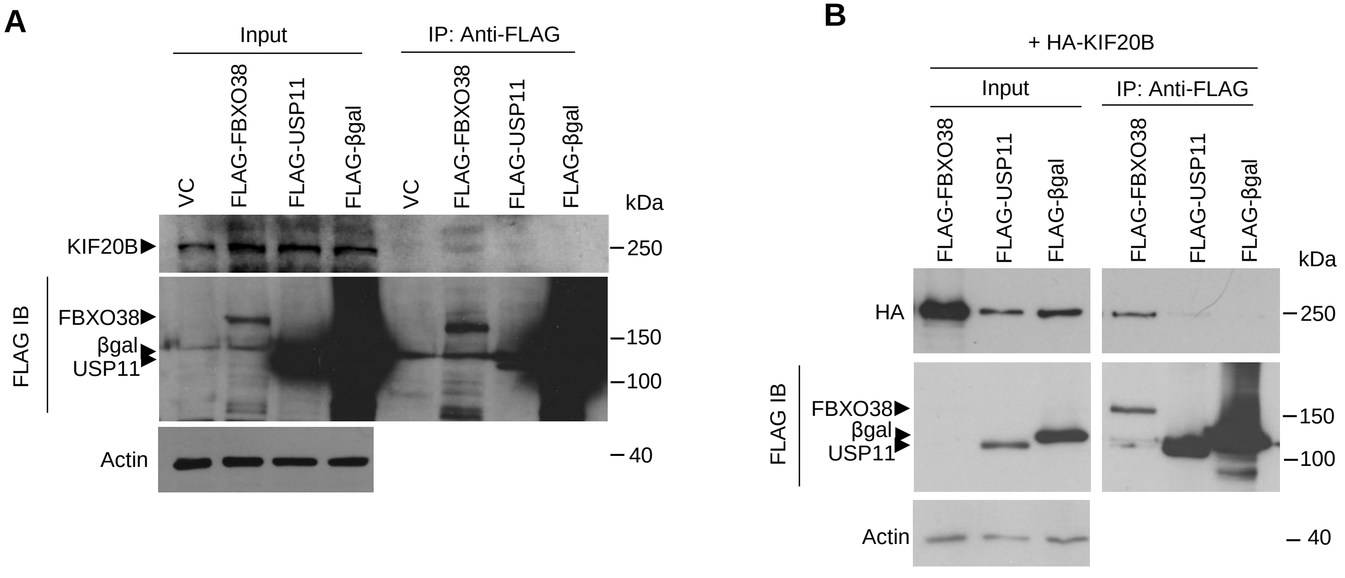

Supplementary Figure S3. Endogenous KIF20B specifically co-immunoprecipitates with FLAG-FBXO38 (A) AGS cells were transfected with a plasmid expressing FLAG-tagged FBXO38, USP11 or βgal or an empty vector control (VC). FLAG-tagged constructs were immunoprecipitated from cell lysates with anti-FLAG resin and recovered proteins were analyzed by Western blotting using antibodies against KIF20B, FLAG and βactin. (B) 293T cells were co-transfected with plasmids expressing HA-tagged KIF20B and the indicated FLAG-tagged protein. FLAG-tagged proteins were immunoprecipitated and recovered proteins were analyzed by Western blotting using antibodies against HA, FLAG and βactin. Bands corresponding to FLAG-FBXO38, USP11 and βgal are indicated with arrow heads. Note that FLAG-FBXO38 expresses at much lower levels than FLAG-USP11 or FLAG- βgal so is not detected in the Input blot at the exposure shown. However the IP lanes confirm that it is expressed and recovered in the FLAG IP. Consistent with results in Fig 4A and 4B, the presence of FLAG-FBXO38 results in increased levels of HA-KIF20B (as seen in the Input lanes).

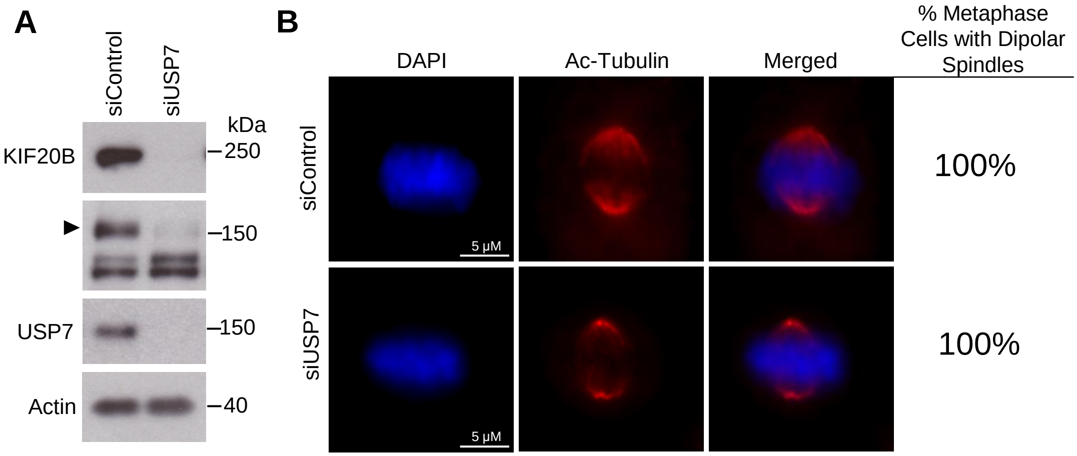

Supplementary Figure S4. USP7 silencing in AGS cells does not induce multipolar spindles. AGS cells transfected with siRNA targeting USP7 (siUSP7) or negative control siRNA (siControl) were either analysed by Western blotting with the indicated antibodies to confirm USP7 silencing (A) or fixed in methanol, stained with an anti-acetylated tubulin antibody and DAPI, then imaged by fluorescence microscopy (B). Cells in metaphase were examined for dipolar and multipolar spindles. A total of 50 metaphase cells were observed for each sample in two independent experiments, but all cells had normal dipolar spindles. Representative images of metaphase cells for siControl and siUSP7 samples are shown.

Legends for Supplementary Tables

Table S1. Complete AP-MS data for FLAG-USP7 and FLAG-β-Gal. All proteins identified in AP-MS experiments with FLAG-USP7 (tab1) and FLAG-β-Gal (tab 2) are shown along with total spectral counts (peptide number), number of unique peptides and % coverage.

Table S2. Complete BioID data for FBXO38. Total peptide counts of all proximal interactions with FLAGBirA*–FBXO38 are shown for each experiment in the raw data tab along with FLAGBirA* negative control values. High confidence interactions specific for FLAGBirA*–FBXO38 are shown in tab 1 (based on SAINT and Bayesian False Discovery Rate (BFDR) scores).

Table S3. KIF20B recovery in BioID experiments with 200 bait proteins. Total peptide counts for KIF20B are shown for two BioID experiments performed with 200 different FLAGBirA* tagged bait proteins. PMIDs references for each experiment are also indicated.
